# Canine Angiostrongylosis in Naturally Infected Dogs: Clinical Approach and Monitoring of Infection after Treatment

**DOI:** 10.1155/2013/702056

**Published:** 2013-12-29

**Authors:** Paola Paradies, Manuela Schnyder, Antonio Capogna, Riccardo Paolo Lia, Mariateresa Sasanelli

**Affiliations:** ^1^Department of Emergencies and Organs Transplantations, Veterinary Internal Medicine Unit, University of Bari, Strada Provinciale per Casamassima Km 3, 70010 Valenzano, Italy; ^2^Institute of Parasitology, Vetsuisse Faculty, Winterthurerstr. 266a, CH-8057 Zürich, Switzerland; ^3^Department of Veterinary Science, Unit of Parasitology, University of Bari, Strada Provinciale per Casamassima Km 3, 70010 Valenzano, Italy

## Abstract

Canine angiostrongylosis is an increasingly reported disease in Europe which can be fatal if left untreated. The wide range of clinical presentation along with the absence of pathognomonic alterations can make the diagnosis challenging; thus any additional information that may provide clues to an early diagnosis may be of value, in order to ensure adequate anthelmintic treatment. Aim of the study was to assess a clinicopathological scoring system associated with natural *Angiostrongylus vasorum* infection diagnosed in canine patients during clinical practice, to clinically and paraclinically monitor infected dogs after treatment, and to monitor the presence of L1 larvae in faecal samples by Baermann's test. Of the total 210 enrolled animals *A. vasorum* infection was diagnosed in 7 dogs. These dogs were clinically and paraclinically investigated and monitored after specific treatment. Further 3 symptomatic dogs were retrospectively included in the monitoring. Results suggest that the computed scoring system can help to increase the clinical suspicion of infection particularly in asymptomatic dogs before the onset of potentially lethal lesions. Data of faecal monitoring suggested that treatment may control parasite burden but be unable to eradicate infection. Thus, a continued faecal monitoring after treatment is advisable for identification of still infected or reinfected dogs.

## 1. Introduction

Canine angiostrongylosis is an increasingly reported disease, possibly due to climatic factors [1], to the presence of foxes acting as parasite reservoir in urban areas [2] or increased travelling of dog owners with their animals [3] which may have facilitated the spread and establishment of the parasite, or, more simply, to more accurate diagnostic methods [4]. In Italy, data on the prevalence of canine angiostrongylosis are scant [5]; however, cases have been recently reported [6–10], suggesting a spread of the infection into previously free areas.

The parasite *Angiostrongylus vasorum* defined as the “great imitator” [11] is responsible for different clinical pictures; anyway the infection is generally associated with respiratory signs, occasionally coupled with neurological and coagulation disorders [12]. The severity of symptoms can greatly vary ranging from asymptomatic to severe forms [7, 11–13]. Laboratory [14–16], radiographic [17, 18], echocardiographic [19–21], and thoracic computer tomographic abnormalities [22, 23] are nonspecific.

The gold standard to achieve the diagnosis still remains the first-stage larvae (L1) detection using the Baermann technique on faecal samples, preferably collected over three consecutive days because of the intermittent larval detection in faeces [24]. Molecular tests on different materials have been developed to improve diagnosis [25–28] and highly sensitive and specific ELISA able to detect circulating antigens as well as specific antibodies in sera sample of infected dogs have also been developed [29, 30].

Treatment of canine angiostrongylosis is performed through oral weekly application of milbemycin oxime for four weeks [31] or a single application of a combination of moxidectin with imidacloprid in a spot-on formulation (Im/Mox) [32], while oral administration of fenbendazole is widely used in different dosages and over several days and appears to be effective but is unlicensed [18]. Infections with *A. vasorum* are of great importance in veterinary medicine, being potentially fatal if left untreated [7, 11, 33]. On the basis of the current literature, the wide range of clinical presentation along with the absence of pathognomonic alterations can make the diagnosis challenging, and it might be hypothesized that a high number of asymptomatic infections are misdiagnosed in endemic areas. Since pathological changes can be present even before clinical signs [16, 32], any additional information that may provide clues to an early diagnosis may be of value, in order to ensure adequate anthelmintic treatment.

Therefore, the aims of this work were to assess a clinicopathological score associated with natural *A. vasorum *infection diagnosed in canine patients; to collect the clinical, laboratory, and imagining data associated with natural *A. vasorum* infection and follow up animals after treatment; and to monitor the presence of L1 in faecal samples evaluated by Baermann's test in a long term followup after treatment.

## 2. Materials and Methods

### 2.1. Study Design, Procedures

From November 2010 to November 2011 a faecal sample was routinely collected directly from the rectal ampulla of owned dogs presented for different problems or for a routine control visit at the Clinical Unit of the Veterinary Faculty of Bari, Italy. In case of empty ampulla, a fresh faecal sample was collected by the owner.

Dogs of all ages, breeds, and living conditions were randomly enrolled in the study, except for animals living exclusively in apartments, which were excluded. Anamnestic data including dog history, living conditions, location, and date and duration of travelling into other regions or abroad as well as the medical history were collected and registered on individual clinical forms, along with data from clinical examination performed at the enrolment. A direct faecal smear was prepared and microscopically examined and the Baermann technique was performed. For patients tested negative but which during the clinical examination showed a clinicopathological score equal to or higher than 3 (see below) the direct faecal smear examination and the Baermann test were also performed on a 3-day faecal pool. Positive dogs were enrolled in the clinical study and treated (Section 2.2). The infection was monitored by means of Baermann's tests (Section 2.3).

For each dog clinicopathological changes suggestive for angiostrongylosis were evaluated and expressed with a score obtained by adding points as follows: presence of signs of coagulation deficits, respiratory disease, neurological signs: 3 points each; laboratory alterations such as anemia (hematocrit < 36%), leukocytosis (leukocytes > 17 × 10³/*μ*L), mild eosinophilia (eosinophils 1.2–1.8 × 10³/*μ*L), hyperglobulinaemia (>4.4 g/dL), or specific increase of *β* globulin (*β*_1_ + *β*_2_ > 1.7 g/dL) and any abnormalities in hemostatic parameters: 1 point each; severe eosinophilia (eosinophils > 1.8 × 10³/*μ*L), lung interstitial/alveolar pattern on thoracic radiographs, pulmonary hypertension at echocardio-Doppler examination: 3 points each. Dogs living in the same environment of diagnosed positive dogs were also enrolled in the study, to which a score of 3 was assigned.

The Baermann technique was performed with 5 g of faeces, with reading at 24 hours. Faecal samples positive for the presence of moving nematode larvae were sent to the Parasitology Unit of the same faculty for morphological identification following McGarry and Morgan [34].

### 2.2. Clinical Followup on Positive Dogs

Each dog positive at faecal examinations was included in the clinical follow-up study. An accurate physical examination and thoracic radiographs, echocardiography, routine laboratory examinations (CBC, biochemical tests, and serum protein electrophoresis), and a coagulation profile were performed in these dogs before anthelmintic treatment as already described [35].

Further 3 symptomatic dogs (dogs 8-9-10) with a previous diagnosis of canine angiostrongylosis on faecal examination were included in the clinical study; their medical records and radiographs were retrospectively reviewed and clinical and paraclinical findings collected.

Asymptomatic dogs were treated with an imidacloprid/moxidectin spot-on formulation (Im/Mox) in a dosage of 25 mg moxidectin/kg BW at T_0_, T_+15_, T_+30_ (Advocate spot-on; Bayer, Animal Health). Symptomatic dogs were treated with fenbendazole 25 mg/kg BW/12 hourly/per os for 21 days (Panacur, Intervet, Animal Health). In symptomatic animals clinical examination was performed once a week until clinical recovery, afterwards twice a month, while asymptomatic animals were reexamined only after the third spot-on treatment. Laboratory, radiographic, and echocardiographic controls were performed after treatment according to owner disposability.

### 2.3. Faecal Monitoring of *A. vasorum* Infections

After treatment, dogs were weekly monitored using the Baermann test on a 3-day faecal pool, both in symptomatic and asymptomatic animals, for one month, afterwards once a month until possible, firstly depending on owner compliance. When infection persisted, a second-line treatment was considered (i.e., Im/Mox spot-on once a month associated with fenbendazole 25 mg/kg BW/per os daily for 21 days).

The study monitoring was stopped when normalization of any clinicopathological alterations and/or 2 consecutive parasitological negative results were reached. The animals were kept under their usual housing conditions and handled and sampled with the owners' consent and with the approval of the Ethical Committee of the Faculty of Veterinary Medicine of the University of Bari (Bari, Italy).

## 3. Results

### 3.1. Score Assignment and Diagnosis of Infection

A total of 210 owned dogs were enrolled in the study. The study population consisted of 97 females and 113 males; 84 were crossbred and 126 purebred animals. The age ranged between 4 months and 16 years (mean 6.2 years) and the weight ranged from 1 to 55 Kg (mean 22.9 Kg). The clinical and pathological abnormalities and scores for dogs included in the study are reported in Table 1. To a total of 74 dogs a clinical score ≥ 3 was assigned.

A direct faecal smear was evaluated in all enrolled dogs (210 samples) and Baermann's test was performed on 162 single samples; in the other 48 dogs the sample volume was not enough to perform the test. A 3-day faecal pool was investigated by means of both direct fecal smear and Baermann's test, in 67 out of 74 dogs showing a clinical score ≥ 3, depending on owner compliance. On the whole, 7 dogs resulted positive for *A. vasorum* larvae at least at one test (see Table 2). *A. vasorum* infection was diagnosed in six dogs from ampulla and/or single fresh faecal samples. The Baermann test performed on the 3-day faecal pool revealed that one further dog was infected. Among the seven positive dogs only one single dog, presented for a routine control visit, had a score < 3 (dog 2). The highest score (11) was reached by a hunting dog (dog 6) presenting severe dyspnoea, while a score of 9 was reached by a dog (dog 7) presented for epistaxis and hyphema. The remaining five positive dogs were normal at clinical examination; nevertheless, they reached a score ≥ 3.

### 3.2. Clinical Followup

Dogs positive for *A. vasorum* infection (*n* = 10) (Table 3) were aged between nine months and 12 years. All dogs were from central-southern Italy and had never moved out of the area. Clinical and paraclinical features at presentation are described in a wider case series study elsewhere [35]. Briefly, the owner complaint was heterogeneous: dyspnea, polypnea, mild/severe coughing, gradual distension of the abdomen, epistaxis, and acute unilateral hyphema. Five cases were presented for routine control visits and one case for specific ocular examination. Among paraclinical alterations the most interesting were a variable increase in *β* globulin fraction at serum protein electrophoresis in all, except for two, dogs and the serpiginous/circular areas of radiopacity revealed at thoracic radiographs in asymptomatic dogs as already described [35].

Fenbendazole was administered in monotherapy in five cases; Im/Mox was administered in monotherapy in the other five cases (Table 3). In general, clinical pictures significantly improved in all symptomatic dogs after 1-2 weeks of treatment and the remission of radiographic abnormalities, when available, was registered at different times.

### 3.3. Faecal Monitoring

Results of faecal monitoring are reported in Table 4. In three dogs a second-line treatment was needed because of the persistence of positive results on fecal samples after 12 weeks from treatment beginning (dog 2 and 3) or because a reverse to positive results was registered during monitoring (dog 5). Fenbendazole 25 mg/kg/day for 21 days associated with Im/Mox was used as second-line treatment. In these three dogs a long term monitoring was performed.

## 4. Discussion

Given the geographical difference with the previously reported case series [9] and prevalence studies [5, 10] from Italy, this study documents the apparent spread of the parasite in this country. The clinicopathological score has been proposed to increase the chances to detect infected dogs. It has been formulated on the basis of the most commonly described clinical and paraclinical alterations associated with *A. vasorum *infection in the literature [4, 15, 17, 20, 21]. Sharing the same environment of infected dogs has been included in the scoring system because it suggests the potential presence of affected gastropods vectors in that area.

Combining the scoring system with faecal tests introduced as routine tests in clinical practice permitted to identify asymptomatic dogs. Diagnosis of angiostrongylosis was performed in all dogs by L1 detection in faecal samples and identification based on morphometric characters and measurements [34]. The occasional detection of L1 in the faeces of dogs referred for a routine control visit suggests that faecal samples can reveal asymptomatic subjects, as previously shown [11]. Our results show that testing three-day fecal pools only partially improved the diagnostic chance to reveal infection, as reported in recent prevalence studies [36, 37].

The reported cases show different clinical presentations associated with the presence of *A. vasorum* in dogs, confirming that angiostrongylosis should be considered as a possible etiological cause of several from mild to severe clinical conditions [11]. As previously described, pathological paraclinical findings have been registered in both symptomatic and asymptomatic dogs. In particular the increase in *β* globulin fraction at serum protein electrophoresis and the serpiginous/circular areas of radiopacity on thoracic radiographs were documented as useful findings that may help to reveal asymptomatic infections [35].

Given its proven efficacy [12, 38], fenbendazole has been used in symptomatic patients, whereas Im/Mox was used for asymptomatic dogs or dogs without life-threatening clinical signs in this study. All dogs treated with fenbendazole (dogs 6–10) resulted negative at Baermann after 1-2 weeks of treatment but a long term monitoring was available only in two dogs (dogs 6, 7), showing negative results. A highly variable response was registered in dogs treated with Im/Mox (dogs 1–5). Two dogs (dogs 1 and 4) reached negative results 1-2 weeks after first spot-on administration and persisted negative for 16 and 20 weeks, respectively. It is reported that larval excretion may continue for over 3 weeks, even if anthelmintic treatment was successful [16]. One case (dog 5) slowly reached negative results (8 weeks after the first spot-on) but it became again positive two times during monitoring, 20 and 24 weeks after the first- and second-line treatment, respectively. The other two cases (dogs 2 and 3) resulted persistently positive for 12 weeks. For these persistently positive dogs, reinfection or coprophagia may be assumed. The last three dogs were treated with a second-line treatment. Cases  2 and 3 achieved negative results slowly (8 and 20 weeks after second-line treatment) making a direct association with treatment efficacy difficult, while they persisted negative in a long term followup. Case  5 showed negative results 4 weeks after second-line treatment but unexpectedly the dog reversed to positive a second time. Reinfection or uncontrolled infection with only partial efficacy treatment is possible, as well as coprophagia leading to false positive results. The question remains open. It may be suggested that anthelmintic treatments may not always completely eliminate adult worms but be able to sterilise them, as shown for *Dirofilaria immitis* infections, possibly leading to a reduced pathogenicity of *A. vasorum* infections. In experimental studies [16] comparing dogs treated with Im/Mox spot-on and untreated dogs, eggs and larvae were only present in untreated dogs. In the same study, larval excretion stopped within 20 days in all 4 treated dogs [16]. However, as evidenced by the same authors, dogs were infected once, whereas in nature dogs can repeatedly ingest infected snails. Oliveira-Júnior and colleagues [24] reported on the larval output of dogs infected and reinfected with *A. vasorum* presenting continuous or irregular pattern in larval output.

The highly variable response to treatment registered in this study suggests that under natural conditions several factors could affect the response to treatment and the diagnosis of *A. vasorum* infections, possibly associated with the host and/or to the parasite biology. Moreover, to monitor the response to treatment, serial tests are needed since the limits of Baermann's tests are known and a single negative result does not mean absence of larval excretion.

## 5. Conclusions

Assessing a clinicopathological score can help to increase the clinical suspicion of infection particularly in asymptomatic dogs before the onset of potentially lethal lesions. Early detection of infected dogs is important because pathological changes may be already present in asymptomatic dogs [32]. Furthermore, the identification of asymptomatic dogs is an important task to reduce the risk of parasite importation in free areas due to trips of animals from endemic areas to free regions [39]. Thus, a move towards routine diagnostic screening in healthy dogs is auspicable. Faecal monitoring after treatment confirmed what was already stated: treatment may control parasite burden but be unable to eradicate infection [40]; thus, continued monitoring after treatment is advisable for identification of still infected or reinfected dogs or for differentiation between infections and coprophagia.

## Figures and Tables

**Table:** (a)

Clinicopathological changes	Dogs with change (*n*)	*A. vasorum* positive dogs (*n*)
Signs of coagulation deficits	2	1
Respiratory disease	30	1
Neurological signs	14	1
Anemia (hematocrit < 36%)	25	1
Leukocytosis (leukocytes >17 × 10^3^/*μ*L)	16	2
Mild eosinophilia (eosinophils 1.2–1.8 × 10^3^/*μ*L)	13	1
Hyperglobulinaemia (>4.4 g/dL)	4	0
Specific increase in *β* globulin (*β*_1_ + *β*_2_ > 1.7 g/dL)	57	4
Any abnormalities in hemostatic parameters	15	1
Severe eosinophilia (eosinophils > 1.8 × 10^³^/*μ*L)	6	2
Lung interstitial/alveolar pattern on thoracic radiographs	3	3
Pulmonary hypertension at echocardio-Doppler examination	1	0
Shared environment with positive dogs	17	1

**Table:** (b)

Score	No. of dogs (of which *A. vasorum* is positive (*n*))
0	77
1	40 (1)
2	19
3	42 (1)
4	22 (1)
5	6 (1)
6	1
7	—
8	1 (1)
9	1 (1)
10	—
11	1 (1)

**Table 2:** Breed, sex (M: male, F: female), age (y: years, m: months), clinical pathological score, and results of diagnostic tests (neg: negative, pos: positive, and nd: not determined) of 7 dogs resulting positive to *Angiostrongylus vasorum* infection.

	Dog	Score	Score single points**	Direct smear	Single sample, Baermann	Three-day pool direct smear	Three-day pool, Baermann
1*	German Shepherd M, 2 y, 32 kg	4	↑WBC, 1 ↑EOS, 3	neg	pos	neg	neg

2*	Crossbred M, 4 y, 31 kg	1	↑*β*_1_ *β*_2_, 1	neg	pos	nd	nd

3	Crossbred sterilized F, 4 y, 31 kg	8	Rx, 3 Environment, 3 ↑*β*_1_ + *β*_2_, 1 ↑EOS, 1	neg	pos	nd	nd

4*	Crossbred sterilized F, 12 y, 26 kg	5	Rx, 3 ↑*β*, 1 ↓AT, ↑fibr., 1	neg	pos	neg	neg

5	Labrador F, 6, 5 y, 29 kg	3	Seizures, 3	pos	nd	neg	pos

6	Breton F, 1 y, 18 kg	11	Dyspnea, 3 Rx, 3 ↑WBC, 1 ↑EOS, 3 ↑*β*, 1	neg	neg	neg	pos

7	Crossbred M, 9 m, 15 kg	9	coagulation deficit signs, 3 Rx, 3 ↓HCT, 1 ↓PLT, 1 ↑*β*_1_ *β*_2_, 1	pos	pos	nd	nd

Total				2/7	*5/6 *	0/4	2/4

*Animals presented for routine control visit.

**See Materials and Methods section.

**Table 3:** Clinical and paraclinical findings at diagnosis in *Angiostrongylus vasorum* naturally infected dogs (*n* = 10) and remission time after specific therapy. The bold data cells indicate dogs that where included in the study “a posteriori.”

Case	1	2	3	4	5	6	7	8	9	10
History and clinical complaint	Routine visit	Routine visit	Routine visit; sporadic coughing	Routine visit; sporadic coughing	Routine visit	Exercise intolerance and cough for 2 months	Epistaxis, hemoptysis cough, and depression for 2 months, inappetence and unilateral hyphema for 2-3 days	**Cough, fever, depression, inappetence for 1 month, several respiratory crises for 2 days**	**Cough for 5 months, progressive abdomen distension for 2 months**	**Decreased vision for 1 year, sporadic coughing**

Clinical findings	Normal	Normal	Normal	Normal	Normal	Expiratory dyspnea	Unilateral hyphema, pale mucous	**Dyspnea, fever**	**Abdomen distention**	**Partial retinal detachment**

Thoracic auscultation	Normal	Normal	Normal	Normal	Normal	Rales and wheezing	Normal	**Muffling of the heart sounds, loud bronchovesicular sound**	**Few rales at the end of inspiration**	**Rales on stimulation of coughing, loud bronchovesicular sound**

Thoracic radiographs	Normal	Circular areas of radiopacity	Circular areas of radiopacity	Circular areas of radiopacity	Circular areas of radiopacity	Diffuse interstitial-bronchial pulmonary pattern, dilatation of the right heart	Diffuse interstitial-bronchial pulmonary pattern, dilatation of right heart	**Moderate bilateral pleural effusion, diffuse interstitial pulmonary pattern, alveolar pattern in the cranial left lobe**	**Diffuse interstitial-bronchial pulmonary pattern, pulmonary artery trunks, dilatation of the right heart**	**Diffuse interstitial pulmonary pattern**

Diagnosis	L1 with Baermann	L1 with Baermann	L1 with Baermann	L1 with Baermann	L1 on direct fecal smear	L1 with Baermann	L1 on direct fecal smear	**L1 in the pleural effusion and faeces**	**L1 on direct fecal smear**	**L1 with Baermann**

Therapy	Im/Mox every 15 days for 3 times	Im/Mox every 15 days for 3 times	Im/Mox every 15 days for 3 times	Im/Mox every 15 days for 3 times	Im/Mox every 15 days for 3 times	Fenbendazole 25 mg/kg, OS q 12 h for 21 days	Fenbendazole 25 mg/Kg, OS, q12 h for 21 days	**Fenbendazole 25 mg/kg, OS q 12 h for 21 days**	**Fenbendazole 25 mg/kg, OS q 12 h for 21 days**	**Fenbendazole 25 mg/kg, OS q 12 h for 21 days**

Clinical recovery	n.a.	n.a.	n.a.	n.a.	n.a.	After 1 week	1 week (ocular problems solved in 2 months)	**After 1 week**	**After 2 weeks**	**After 2 weeks**

Remission of radiographic signs	—	2 months	N.K.	3 months	2 months	5 months	N.K.	**N.K.**	**N.K.**	**N.K.**

*n.a: not applied; **N.K.: not known.

**Table 4:** Results of faecal monitoring by Baermann's test. The asterisks show the passage to a second-line treatment (fenbendazole 25 mg/kg/os/daily for 21 days plus Im/Mox spot-on once a month) for dogs 2, 3, and 5.

Dog number	1	2	3	4	5	6	7	8	9	10
Faecal monitoring										
1 week	nd	+	+	−	nd	−	+	+	+	−
2 weeks	−	+	nd	+	+	−	−	−	+	−
3 weeks	nd	+	nd	−	+	−	−	−	−	−
4 weeks	−	+	+	−	nd	nd	−	−	−	−
6 weeks	nd	+	+	−	+	nd	nd			
8 weeks	nd	+	+	−	−	nd	nd			
12 weeks	−	+*	+*	nd	−	nd	nd			
16 weeks	−			−	−	−	−			
20 weeks				−	+*	nd				
24 weeks						−				
Second-line treatment		Fenb. + Im/Mox	Fenb. + Im/Mox		Fenb. + Im/Mox					
Faecal monitoring										
2 weeks		+	nd		+					
3 weeks		nd	+		nd					
4 weeks		+	nd		−					
6 weeks		+	+		nd					
8 weeks		+	−		−					
20 week		−	−		nd					
24 week		nd	nd		+					
28 week		−	−		−					
44 week		−	−							
